# Supplementary material for: Seminoma and Embryonal Carcinoma Footprints Identified by Analysis of Integrated Genome-Wide Epigenetic and Expression Profiles of Germ Cell Cancer Cell Lines
Source: PLoS One. 2014 Jun 2;9(6):e98330. doi: 10.1371/journal.pone.0098330 (PMC4041891; doi:10.1371/journal.pone.0098330)
Supplement: File S1 — ZIP file containing DMRforPairs output for significant regions. Please start from the html files. (ZIP) [file pone.0098330.s008.zip › figures/36984.pdf]

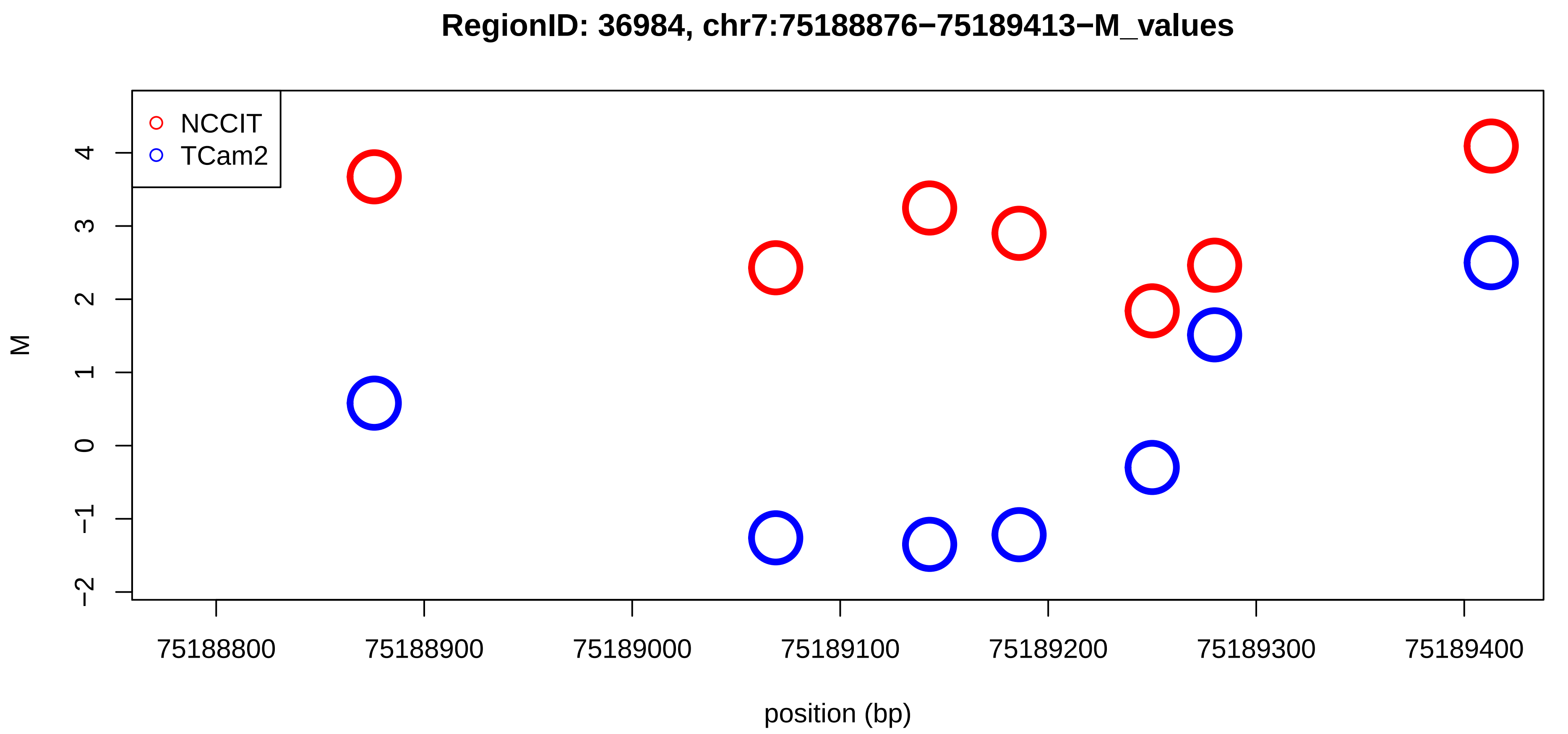
RegionID: 36984, chr7:75188876−75189413−M_values
NCCIT
TCam2
M
position (bp)
75188800
75188900
75189000
75189100
75189200
75189300
75189400

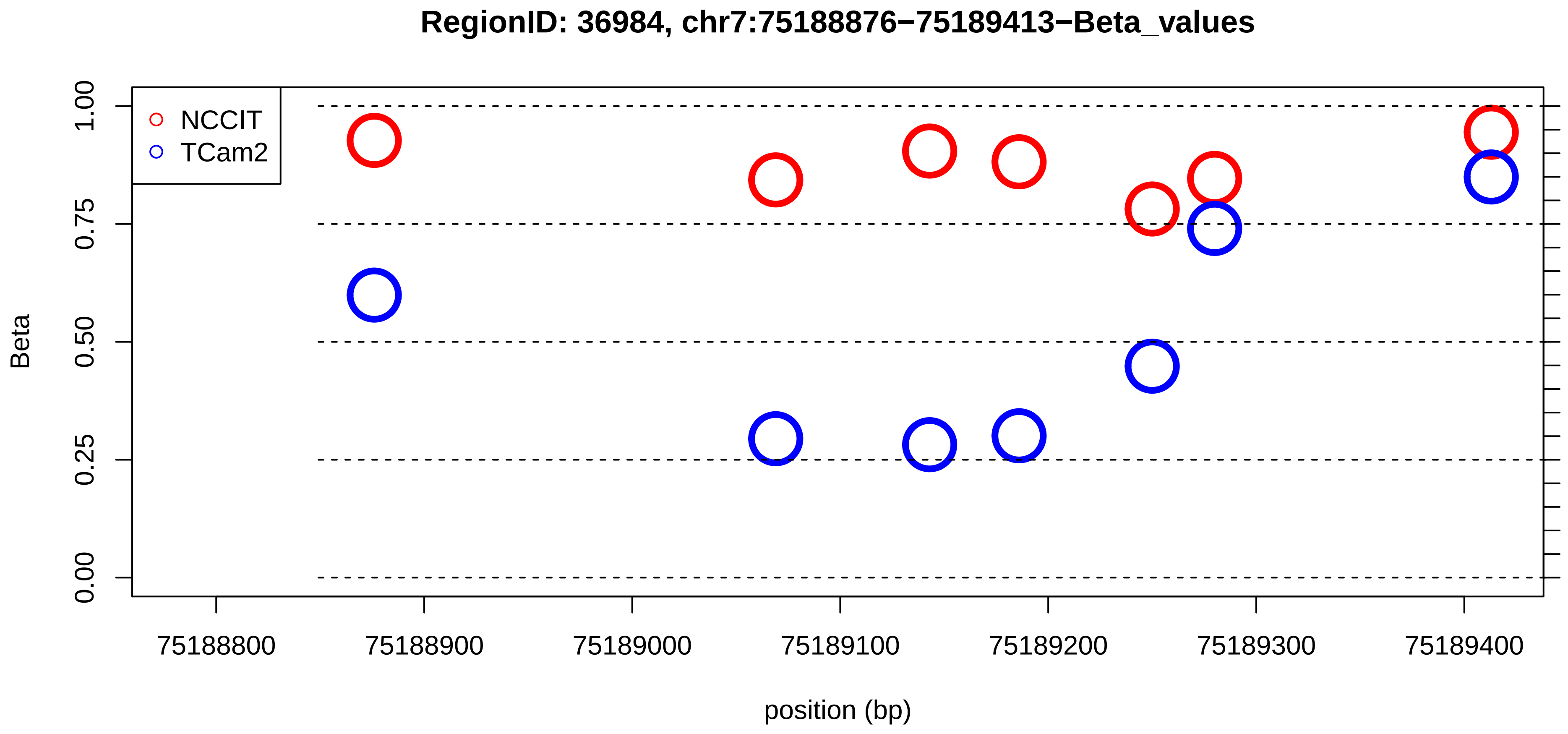
RegionID: 36984, chr7:75188876−75189413−Beta_values
NCCIT
TCam2
Beta
position (bp)
75188800
75188900
75189000
75189100
75189200
75189300
75189400
